# Ambient Effect Filtering Using NLPCA-SVR in High-Rise Buildings

**DOI:** 10.3390/s20041143

**Published:** 2020-02-19

**Authors:** Xijun Ye, Yingfeng Wu, Liwen Zhang, Liu Mei, Yunlai Zhou

**Affiliations:** 1 School of Civil Engineering, Guangzhou University, Guangzhou 510006, China; xijun_ye@gzhu.edu.cn (X.Y.); lwzhang@gzhu.edu.cn (L.Z.); 2 Guangdong Provincial Key Laboratory of Durability for Marine Civil Engineering, Shenzhen University, Shenzhen 518060, China; meiliu@szu.edu.cn; 3 Department of Civil and Environmental Engineering, The Hong Kong Polytechnic University, Hong Kong 999077, SAR, China

**Keywords:** ambient effects, modal frequency, Guangzhou New TV Tower, nonlinear principal component analysis, support vector regression

## Abstract

The modal frequencies of a structure are affected by continuous changes in ambient factors, such as temperature, wind speed etc. This study incorporates nonlinear principal component analysis (NLPCA) with support vector regression (SVR) to build a mathematical model to reflect the correlation between ambient factors and modal frequencies. NLPCA is first used to eliminate the high correlation among different ambient factors and extract the nonlinear principal components. The extracted nonlinear principal components are input into the SVR model for training and predicting. The proposed method is verified by the measured data provided in the Guangzhou New TV Tower (GNTVT) Benchmark. The grid search method (GSM), genetic algorithm (GA) and fruit fly optimization algorithm (FOA) are applied to determine the optimal hyperparameters for the SVR model. The optimized result of FOA is most suitable for the NLPCA-SVR model. As evaluated by the hypothesis test and goodness-of-fit test, the results show that the proposed method has a high generalization performance and the correlation between the ambient factor and modal frequency can be strongly reflected. The proposed method can effectively eliminate the effects of ambient factors on modal frequencies.

## 1. Introduction

### 1.1. Background

Accurate identification of the modal frequency of structures is an important task for structural health monitoring (SHM). The modal frequency, determined by the structural stiffness and mass, can be affected by different ambient factors, such as temperature, wind direction and wind power, and so on. Ambient factors, especially temperature, can cause changes in modal frequencies even greater than the changes caused by structural damage [1].Since then, many researchers have studied temperature-induced variations in natural frequency of structures. In the past two decades, bridges have been paid more attention than other types of structures, possibly because bridges are directly exposed to the ambient environment. On the basis of two-year continuous dynamic monitoring data from two bridges under normal operating conditions, Hu et al. [2] found that temperature has a primary effect on the variations of modal frequencies in a nonlinear manner. The annual maximum relative variation of frequency estimates is in the14–20.6% range for the 12 modes analyzed, which would mask the subtle changes induced by structural damage. Deng et al. [3] monitored the Yunyang Suspension Bridge with a 1490-m main span for a period of 10 months. The first six modal frequencies experienced about 2% variation, as the ambient temperature of the steel bridge varied from −5 to +50 °C. In recent years, as more and more high-rise buildings have been built, variations in frequencies of high-rise structures have attracted more and more studies as extensively as bridge structures. In the field monitoring of a 17-story steel frame building, Nayeri et al. [4] found that there is a strong correlation between and temperature and frequencies, whereas frequency variations lagged behind temperature variations by a few hours. Based on the one-year monitoring data of a 22-story reinforce concrete (RC) building, Yuen and Kuok [5] found that the first three frequencies increased with an increase in ambient temperature, which was opposite to their analytical results. During a 24-h period of field monitoring, Faravelli et al. [6] studied the variations in frequencies of the 600-m-tall Guangzhou New TV Tower. By monitoring the Shanghai Tower (with a height of 632 m) for 12 h, Zhang et al. [7] found that the natural frequency has an ascending trend with increasing temperature, with slight decreases with an increase of humidity. Domarie and Sabia [8] studied the variations of modal frequencies based on the continuous monitoring data over a year of a high bell tower. The frequency values tended to vary in different trend in different temperature period. Furthermore, such a variation was smooth for the bending modes, while it showed as abrupt for the torsional modes. Ni et al. [9] carried out a field test to measure a tall building for 48 h to investigate the performance variance and the distribution of the modal parameters. Wu et al. [10] conducted a long-term monitoring and condition evaluation of an office building. A crucial observation from this assessment is that the percentages of frequency variation in three months for most of the identified modes were beyond 10%.Although in the normal temperature range, the change of frequency is usually at the level of a few percentages, which can be more severe than the change caused by structural damage. In order to avoid false condition evaluation, the relation between temperature and structural modal parameters should be established so that the temperature effect can be eliminated in condition evaluation.

In eliminating the effect of ambient factors on modal parameters, the key is to build a mathematical model that can accurately reflect the intrinsic relation between modal parameters and ambient factors. Many methods have been applied to eliminate the influence of ambient factors on modal parameters. The commonly used methods are the Bayesian framework [11,12], time series analysis [13,14,15] and artificial neural network (ANN) [16,17,18]. Behmanesh et al. [11] presented a hierarchical Bayesian framework in the absence of noise or model discrepancies to accurately identify parameters subjected to external actions. Jesus et al. [12] applied the Bayesian framework to the structural identification of a long suspension bridge by considering temperature and traffic load effects. Liu et al. [15] established a structural health monitoring (SHM) benchmark database for a prestressed concrete box girder bridge, and a linear regression model between the first three modal frequencies and temperatures was built based on the monitored data. Li et al. [16] studied the dependence of the modal frequency, modal shape and damping ratio on temperature and wind speed. For certain modes, temperature was the most significant environmental factor that accounted for the variation of damping ratios, while for other modes, wind velocity was the significant factor. Shan et al. [17] applied three regression-based numerical models, including multiple linear regression (MLR), back-propagation neural network (BPNN), and support vector regression (SVR) to capture the relations between modal frequencies and temperature distributions from measurements of a concrete beam during a period of 40 days. Teng et al. [18] conducted the continuous dynamic monitoring of a bridge and applied ANN to remove the temperature effect on modal frequencies so that a health index can be constructed under operational conditions.

### 1.2. Motivation and Objectives

The support vector machine (SVM), proposed by Vapnik in 1995, is a new pattern recognition method implementing the structural risk minimization inductive principle to obtain a strong generalization performance with limited samples. Support vector regression (SVR) is the most common application form of the SVM. Instead of minimizing the observed training error, SVR attempts to minimize the generalization error to achieve a strong generalized performance. In the SVR model, the key is the determination of hyperparameters, which is related to the generalization and prediction performance [19,20,21]. Based on the principle of structural risk minimization, SVR, a global optimization method, shows a strong generalization performance for limited samples and can effectively solve the nonlinear problem. SVR can model the correlation of the ambient factor-modal frequency with limited samples.

In addition, temperature is generally considered to be the most important factor that affects the structural modal frequency, but some studies have found that wind speed changes will cause structural aerodynamic stiffness changes [16], thus resulting in structural modal frequency changes. Moreover, a strong correlation among different ambient factors will negatively impact the generalization performance of the SVR model, thereby resulting in low prediction accuracy. Principal component analysis (PCA) is a multivariate analysis technique that is used to simplify the complexity of high-dimensional data while retaining their trends and patterns. The feature vector of the original data vector can be extracted by linear transformation. However, the changing patterns of ambient factors do not satisfy a linear relationship in statistics. Nonlinear principal component analysis (NLPCA) is the nonlinear expression of standard PCA and can reduce the observed variables to a series of uncorrelated principal components. Therefore, NLPCA can be applied to solve the nonlinear problem and will describe the data with greater accuracy than PCA [22,23].

By considering multiple ambient factors (temperature, wind speed and wind direction), this study proposed an algorithm consisting of NLPCA and SVR to model the correlation between ambient factors and modal frequencies. The monitored data of the GNTVT Benchmark is used to verify the generalization performance and reliability.

## 2. Fundamentals of NLPCA-SVR

### 2.1. NLPCA

**Figure 1:**
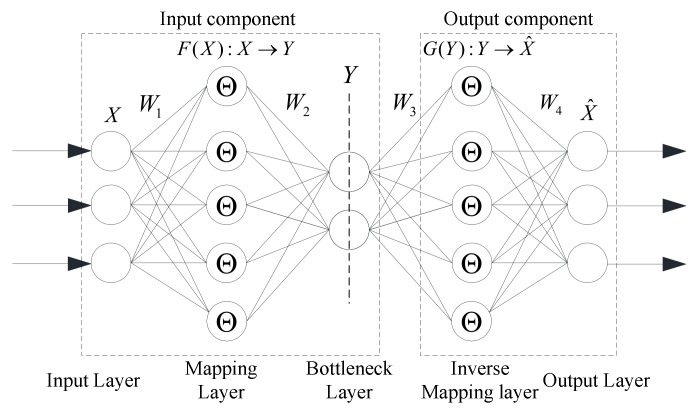
NLPCA network structure based on AANN.

In Figure 1, NLPCA can be expressed as an auto-associative neural network (AANN). AANN is a feed forward neural network with the same number of output and input. The classical network of AANN consists of two parts: the input component and output component [24]. In this paper, ambient factor data are processed by NLPCA.

The input component consists of an input layer, mapping layer and the bottleneck layer. When the original data $X\in R^{n}$ are input, a series of compressions and mixtures in the mapping layer are conducted on $X\in R^{n}$. The feature vector $Y\in R^{m}$ (*m* < *n*) is output in the bottleneck layer.

A linear transfer function is used in the input layer and the bottleneck layer. As a nonlinear transfer function is used in the mapping layer, according to the Cybenko theorem, a nonlinear equation, such as Y=F(X) and $\hat{X}=G(Y)$, can be expressed by a well-trained typical AANN with a hyperbolic function. As the bottleneck layer is the output of the input component, the output vector $Y\in R^{m}(m<n)$ can be expressed by the input vector *X*, weight matrix *W*_1_, *W*_2_ and bias vector value *b*_1_, *b*_2_,

(1) $$Y=W_{2}\Theta (W_{1}X+b_{1})+b$$

where Θ is the hyperbolic function. A suitable function is the sigmoid: $\Theta =\frac{1}{1+e^{-x}}$.

(2) The output component consists of the inverse mapping layer and the output layer. In this part, the linear transfer function is used in the output layer, and the hyperbolic function is used in the inverse mapping layer. The feature vector $Y\in R^{m}$ (*m* < *n*) is regarded as the input of this component. Through the inverse mapping layer, the reconstructed output data $\hat{X}$ are obtained in the output layer, expressed by the vector $Y\in R^{m}$ (*m* < *n*), weight matrix *W*_3_, *W*_4_ and bias vector value *b*_3_, *b*_4_,

(2) $$\hat{X}=W_{4}\Theta (W_{3}Y+b_{3})+b_{4}$$

where Θ is the hyperbolic function. A suitable function is the sigmoid: $\Theta =\frac{1}{1+e^{-x}}$.

In this procedure, the key problem is to accurately define the nonlinear functions *F*(X) and G(Y). In this study, BPNN with a hyperbolic function, an unsupervised learning algorithm, is used to train the function between the input and output vectors. An objective function, shown in Equation (3), is used to measure the loss of data information in the entire transformation procedure.

(3) $$J=\frac{1}{2}E\left[{\left\|X-\hat{X}\right\|}^{2}\right]$$

To determine the nonlinear functions *F*(*X*) and *G*(*Y*), Equation (3) is solved by the gradient-based optimization algorithm to find the best values for the weight and the bias vectors.

### 2.2. Support Vector Regression (SVR)

In this study, a radial bias function (RBF) is used to build the SVR model, and a K-fold cross-validation for combining the grid search method (GSM), genetic algorithm (GA) and fruit fly optimization algorithm (FOA) are applied to determine the hyperparameters of the SVR model.

For a set of training samples, S=[(x_1_,y_1_),(x_2_,y_2_),…,(x_m_, y_m_)] $\in R^{n}\times R$, where *x_i_* is an input vector of n dimension, *y_i_* is the corresponding output vector, and m is the number of samples. The objective of the SVR model is to find a linear regression function *f*(*x*) to minimize the expected risk *R*(*f*). The expected risk follows Equation (4).

(4) $$R\left[f\right]=\int l\left(y-f\left(x\right)\right)dP\left(x,y\right)$$

where *l*(g) denotes the loss function determining how to penalize estimation errors and P(x,y) is probability distribution of the training sample.

To fulfill the stated goal, SVR considers the following linear estimation function

(5) $$f(x)=\langle w,x\rangle +b$$

where w means weight vector; b means the bias constant; and   means inner product.

**Figure 2:**
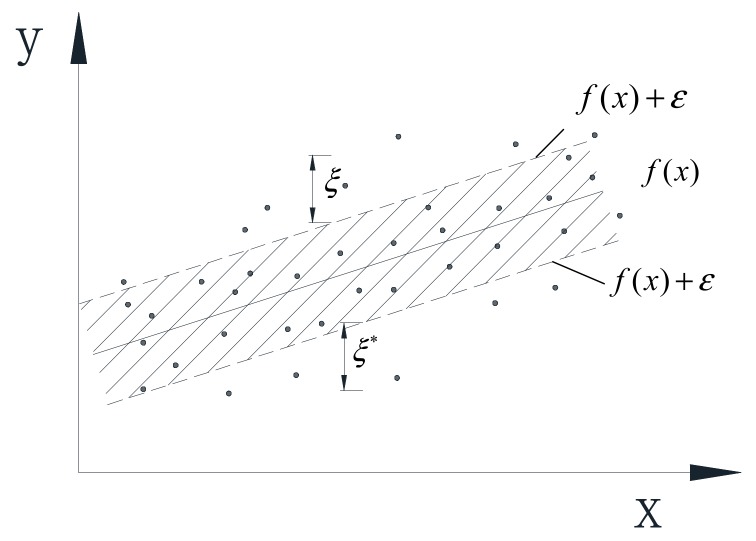
Schematic of the ε-insensitive loss function.

Quadratic loss functions, Huber loss functions, and ε-insensitive loss functions are commonly used as loss functions of SVR model. As the SVR model based on ε-insensitive loss function can reach higher calculation accuracy with less support vectors, it can greatly reduce the computation. In this study, the ε-insensitive loss function is used as the loss function. In Figure 2, only the points outside the shaded region contribute to the cost, as the deviations are penalized in a linear fashion.

The ε-insensitive loss function can be expressed as

(6) $$l\left(f\left(x\right)-y\right)=\left\{\begin{array}{ll} \left|f\left(x\right)-y\right|-\varepsilon & \left|f\left(x\right)-y\right|\geq \varepsilon \\ 0 & otherwise \end{array}\right.$$

where ε represents the radius of the tube located around the regression function *f*(*x*), as illustrated in Figure 2.

With limited samples, the structural risk function R_reg_(*f*) can reduce the overfitting effect in the training process and improve the generalization performance of the SVR model

(7) $$R_{reg}\left(f\right)=R_{emp}\left(f\right)+\frac{1}{2}{\left\|w\right\|}^{2}=\frac{1}{m}\sum_{i=1}^{m}l\left(y_{i}-f\left(x_{i}\right)\right)+\frac{1}{2}{\left\|w\right\|}^{2}$$

where $R_{emp}\left(f\right)=\frac{1}{m}\sum_{i=1}^{m}l\left(y_{i}-f\left(x_{i}\right)\right)$ is the empirical risk function; ${\left\|w\right\|}^{2}=\langle w,w\rangle$ is the Euclidean norm that represents the factors related to the complexity of the model. *l*(*y_i_*-f(*x_i_*)) is the loss function.

Substituting Equation (6) into Equation (7) and solving Equation (7) is equivalent to solving the optimization problem as follows.

(8) $$\left\{\begin{array}{l} \min\frac{1}{2}{\left\|w\right\|}^{2}+C\sum_{i=1}^{m}\left(\xi_{i}+\xi_{i}^{*}\right)\\ s.t\left\{\begin{array}{l} y_{i}-\langle w_{i},x_{i}\rangle -b\leq \varepsilon +\xi_{i}\\ \langle w_{i},x_{i}\rangle +b-y_{i}\leq \varepsilon +\xi_{i}^{*}\\ \xi_{i},\xi_{i}^{*}\geq 0 \end{array}\right. \end{array}\right.$$

where the positive constant C determines the trade-off between the flatness of *f*(*x*) and the amount up to which deviations larger than ε are tolerated; $\xi_{i}^{*}$, $\xi_{i}$ are slack variables.

The dual formulation provides the key for SVR to nonlinear functions. The standard dualization method utilizing Lagrange multipliers is described as follows:

(9) $$\begin{array}{l} L=\frac{1}{2}{\left\|w\right\|}^{2}+C\sum_{i=1}^{m}\left(\xi_{i}^{*}+\xi_{i}\right)-\sum_{i=1}^{m}\alpha_{i}\left(\xi_{i}^{*}+\varepsilon -y_{i}+\langle w,x_{i}\rangle +b\right)\\ -\sum_{i=1}^{m}\alpha_{i}^{*}\left(\xi_{i}^{*}+\varepsilon +y_{i}-\langle w,x_{i}\rangle -b\right)-\sum_{i=1}^{m}\left(\eta_{i}\xi_{i}+\eta_{i}^{*}\xi_{i}^{*}\right) \end{array}$$

where *L* is the Lagrangian and $\alpha_{i},\alpha_{i}^{*},\eta_{i},\eta_{i}^{*}$ are Lagrange multipliers.

According to the KKT (Karush–Kuhn–Tucker) conditions

(10) $$\frac{\partial L}{\partial b}=0\Rightarrow \sum_{i=1}^{m}\left(\alpha_{i}^{*}-\alpha_{i}\right)=0$$

(11) $$\frac{\partial L}{\partial w}=0\Rightarrow w-\sum_{i=1}^{m}\left(\alpha_{i}^{*}-\alpha_{i}\right)x_{i}=0$$

(12) $$\frac{\partial L}{\partial \xi }=0\Rightarrow C-\alpha_{i}-\eta_{i}=0$$

(13) $$\frac{\partial L}{\partial \xi_{}^{*}}=0\Rightarrow C-\alpha_{i}^{*}-\eta_{i}^{*}=0$$

Substituting Equations (10)–(13) into Equation (9) yields dual optimization problems.

(14) $$\max\left\{\begin{array}{l} -\frac{1}{2}\sum_{i,j=1}^{m}\left(\alpha_{i}-\alpha_{i}^{*}\right)\left(\alpha_{j}-\alpha_{j}^{*}\right)\langle x_{i},x_{j}\rangle -\varepsilon \sum_{i=1}^{m}\left(\alpha_{i}+\alpha_{i}^{*}\right)+\sum_{i=1}^{m}y_{i}\left(\alpha_{i}-\alpha_{i}^{*}\right)\\ s.t\left\{\begin{array}{l} \sum_{i=1}^{m}\left(\alpha_{i}-\alpha_{i}^{*}\right)=0\\ \alpha_{i},\alpha_{i}^{*}\in \left[0,C\right] \end{array}\right. \end{array}\right.$$

Equation (11) can be expressed as

(15) $$w=\sum_{i=1}^{m}\left(\alpha_{i}-\alpha_{i}^{*}\right)x_{i}$$

Substituting Equation (15) into Equation (5), Equation (5) can be rewritten as

(16) $$f\left(x\right)=\sum_{i=1}^{m}\left(\alpha_{i}-\alpha_{i}^{*}\right)\langle x_{i},x_{j}\rangle +b$$

where the sample data in $\alpha_{i}-\alpha_{i}^{*}$, does not equal zero, are defined as the support vector.

In transforming the training patterns *x_i_* into a higher dimension feature space by a nonlinear map Φ, a nonlinear SVR model can be obtained. Then, the linear regression is applied to the training patterns *x_i_* in this feature space. Once the nonlinear SVR model is built, <*x_i_*, *x_j_*> can be replaced by the kernel function *K*(*x_i_*,*x_j_*). The nonlinear regression function can be expressed as

(17) $$f\left(x\right)=\sum_{i=1}^{m}\left(\alpha_{i}-\alpha_{i}^{*}\right)\left(\Phi \left(x_{i}\right)\cdot \Phi \left(x\right)\right)+b=\sum_{i=1}^{m}\left(\alpha_{i}-\alpha_{i}^{*}\right)K\left(x_{i},x\right)+b$$

In recent years, some studies [25,26] have proved that SVR based on Radial Bias Function (RBF) has good fitting and generalization performance. Through suitable selection of kernel width σ, RBF can be applied to samples with arbitrary distribution. It has been widely used in support vector regression analysis. The RBF function applied to construct SVR model in this paper can be expressed as

(18) $$K\left(x_{i},x_{j}\right)=\exp\left(-\frac{{\left\|x_{i}-x_{j}\right\|}^{2}}{2\sigma^{2}}\right)$$

where σ is the kernel width.

### 2.3. Flowchart

**Figure 3:**
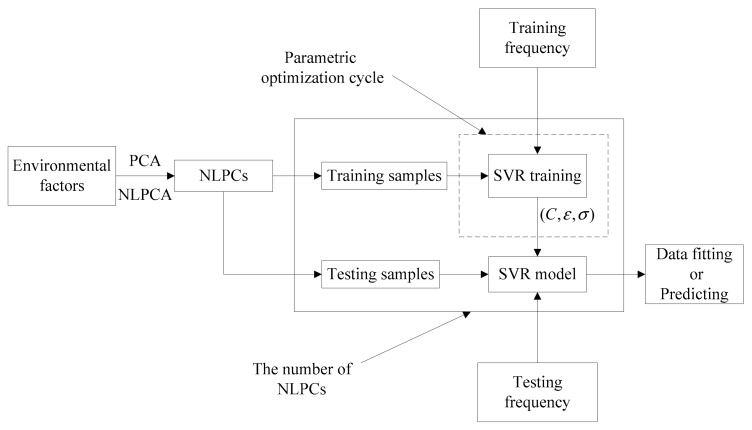
Flowchart of the NLPCA-SVR.

Figure 3 shows the flowchart of the proposed method. First, PCA is used to extract the main feature vectors according to the cumulative variance contribution rate (CVCR). Nonlinear principal components (NLPCs) of the original measured ambient data are extracted by NLPCA, which can eliminate the correlation of different variables. Finally, the extracted NLPCs are used in the SVR model for training.

## 3. Modal Identification of GNTVT

### 3.1. Introduction of the GNTVT Benchmark

**Figure 4:**
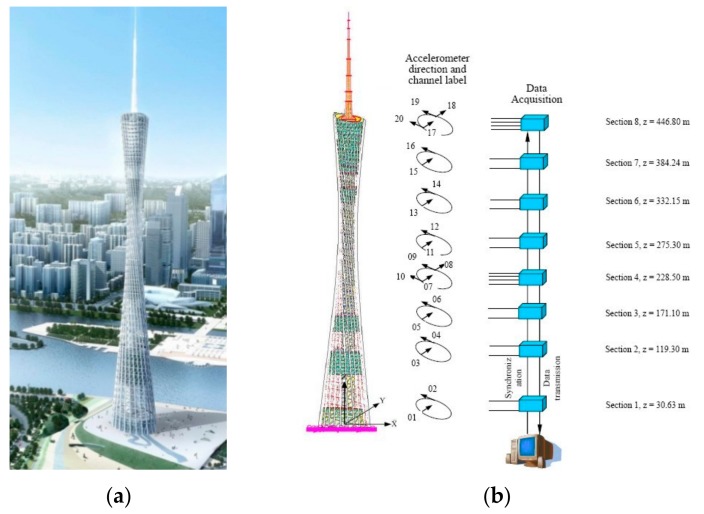
Guangzhou New TV Tower (GNTVT): (**a**) Description of GNTVT; (**b**) SHM scheme.

The Guangzhou New TV Tower (GNTVT) is a 610 m high tube-in-tube structure consisting of a 454 m main tower and a 156 m antenna (Figure 4a), completed in 2010 in Guangzhou, China. On this TV tower, a long-term SHM system for both in-construction and in-service real-time monitoring (Figure 4b) are designed and installed. A GNTVT SHM Benchmark is released [27], 24-h continuous monitoring data for acceleration, temperature, wind direction and wind speed, are measured by the SHM system.

### 3.2. Modal Identification

**Figure 5:**
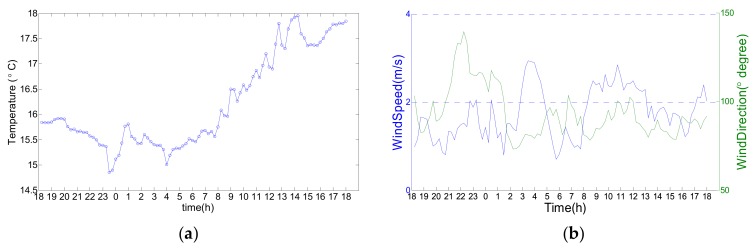
Ambient factors in the GNTVT Benchmark: (**a**) Temperature data; (**b**) Wind speed and wind direction data.

The measured data provided by the GNTVT Benchmark are introduced as follows: the data last for 24 h, from 18:00 on 19 January 2010, to 17:00 on 20 January 2010. The sampling frequency of the acceleration is 50 Hz, the sampling frequency of the temperature is 1/60 Hz, and the sampling frequencies of the wind speed and wind direction are 50 Hz. The measured data are shown in Figure 5. The temperature varies from 14 °C to 18 °C, and the wind speed varies from 0 to 4 m/s.

**Figure 6:**
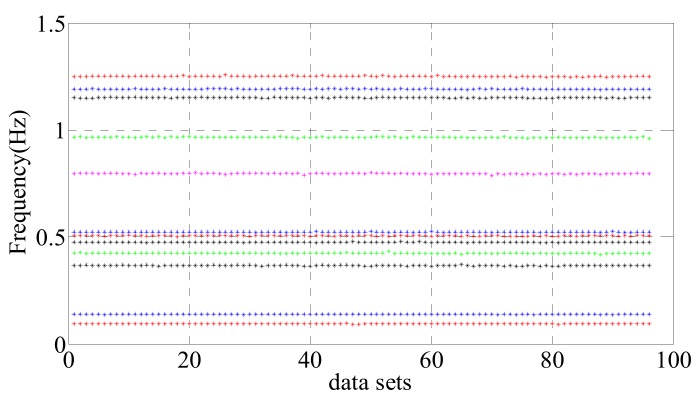
Ninety-sixsets of modal frequencies identified by Natural Excitation Technique/Eigensystem Realization Algorithm (NExT-ERA).

The Natural Excitation Technique/Eigensystem Realization Algorithm (NExT/ERA) is applied to identify the first 12 modal frequencies of GNTVT by Ye et al. [28], as shown in Figure 6. The data for each hour are divided into four groups (15 min each); there are 96 groups of data in total for 24 h. The modal frequency shows a negative correlation with the ambient temperature. Temperature is generally considered to be the most important factor affecting the structural modal frequency, but some studies found [16] that although temperature is the main factor affecting the change of structural modal frequency, the change of wind speed will cause the change of structural aerodynamic stiffness, resulting in the structural modal frequency changes. In this study, the NLPCA-SVR model is applied to eliminate the effect of ambient factors on the modal frequency.

## 4. Data Model

### 4.1. Data Preprocessing

Since there are only 96 data samples, the first three-quarters of the samples are used as the training samples (72 samples), and the remaining samples are used as the testing samples (24 samples). Before training the SVR model, the training samples and test samples were normalized to [−1, 1]. Two different models are compared to each other to verify the generalization performance and computational efficiency. One is the NLPCA-SVR model built from the ambient data processed by NLPCA, and the other is the OriginalData-SVR model built from the original measured ambient data. The training process of the 1st modal frequency is taken for instance.

**Figure 7:**
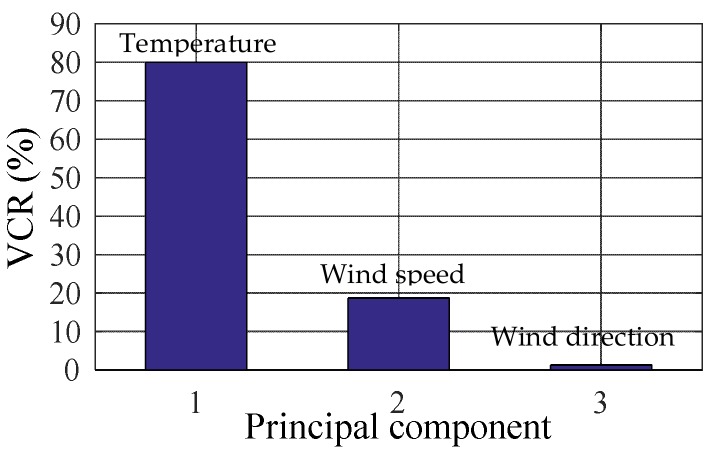
Variance contribution rates of different components.

Temperature, wind speed and wind direction are the ambient effects monitored in this Benchmark. First we use PCA to extract the main features of the ambient factors. Figure 7 shows the cumulative variance contribution rate (CVCR) of each principal component obtained by PCA. The main feature vectors are decided by the cumulative variance contribution rate. Generally, the principal components corresponding to the CVCR of 85–95% are taken as the main feature vectors. In this model, the CVCRs of the first two components reach 98.73%, which means the main feature vectors of the ambient factors.

### 4.2. NLPCA-SVR Model

NPLCA is applied to extract the NLPCs of the original measured ambient data. The NLPCA network topology is set as 3-5-3-5-3 with 1000 iterations. Table 1 shows the correlation coefficients between the ambient factors. The absolute value of the correlation coefficient of the original measured ambient factors to each other is greater than two, while the correlation coefficient of the different NLPCs to each other is close to zero.

#### Optimization of hyperparameters

Once the loss function and kernel function are determined, the SVR model selection is equivalent to the hyper-parameters determination. In the SVR model, the ε-insensitive loss function and RBF kernel function are selected. The hyper-parameter refers to the positive constant C, the insensitive coefficient ε and the kernel width σ. In this study, GSM, GA and FOA are applied to determine the best combination of the insensitive coefficient, positive constant and kernel width for the SVR model.

1. GSM

**Figure 8:**
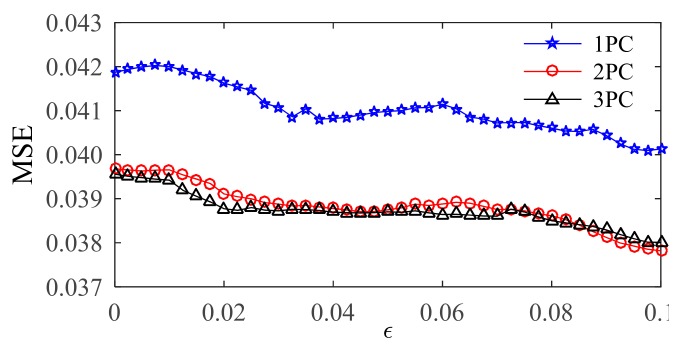
MSE value determined by GSM.

GSM is a practical data search method suitable for parallel searches of multidimensional arrays from different directions at the same time [29]. Figure 8 shows the calculation process when different numbers of NLPCs are used as inputs. Combining the K-fold cross-validation with GSM, the specific implementation process of the algorithm is as follows:

(1) With a step size of ε_step_ = 0.0025, the insensitive coefficient ε is set to vary from 0 to 0.1. For each ε, the best combination of (C, σ) is determined by steps (2) to (4);
(2) The step sizes of the penalty parameters C and kernel functions σ are both set as 0.5. With a step size of 0.5, C and σ are both set to vary from 2.8 to 28;
(3) A different combination of (C, σ) is substituted into the SVR model in turn. K-fold cross validation is applied to reduce the influence of the data set partitioning differences. The training data set is randomly divided into K groups of subsets (approximately equal sizes) that are not included in each other. (K-1) groups of the subset are set as the training sets, and the others are set as the testing sets. This process is repeated K times so that each subset is tested, the mean square error (*MSE_cv_*) of k times is obtained
  (19) $$MSE_{CV}=\frac{1}{m}\sum_{i=1}^{m}\left(y_{i}-{\bar{y}}_{i}\right)$$
  where ${\bar{y}}_{i}$ and *y_i_* are the training and expected output values in the SVR model, respectively, and m is the training sample number;
(4) For different combinations of (C, σ), their MSEs are plotted with contour lines to obtain a contour map. According to this contour map, the best combination of (C, σ) is determined by $E=\min\left\{MSE(\lambda )\left|\lambda =1,2,\cdots ,p\times q\right.\right\}$;
(5) For each ε_step,_ the best combination of (ε_step_, C, σ) can obtain E. The optimal combination (ε, C, σ) can be determined by $E=\min\left\{E(i)\left|i=1,2,\cdots ,\right.\frac{\left(\varepsilon_{\max}-\varepsilon_{\min}\right)}{\varepsilon_{step}}\right\}$.

2. GA

GA is a stochastic global optimization and search method [30]. The parameter settings for GA are listed as follows:

(1) The ranges of the three parameters (C, ε, σ) are set as [0,200], [0,1], [0,200], respectively.
(2) The generation number, population number, crossover probability, and mutation probability are set to 200, 20, 0.7, and 0.05, respectively.
(3) K-fold cross-validation is applied in GA. MSE_CV_ is set as the fitness function, expressed as

(20) $$\min\left(MSE_{CV}\right)=\min\left(\frac{\sum_{i=1}^{m}{\left(y_{i}-{\bar{y}}_{i}\right)}^{2}}{m}\right)$$

where *m* is the training sample number and *y_i_* and ${\bar{y}}_{i}$ are the expected output value and training output value in the SVR model, respectively.

**Figure 9:**
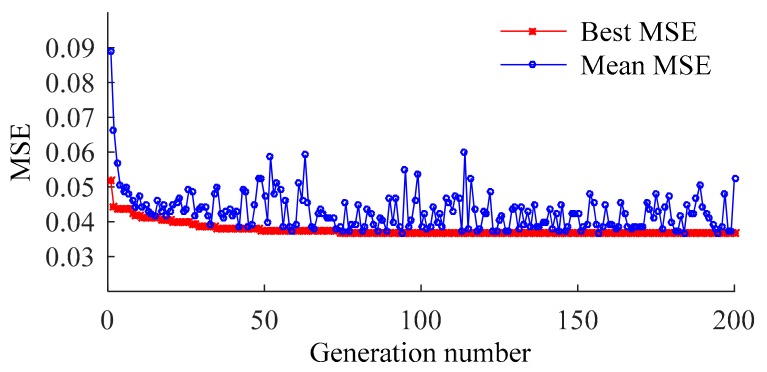
*MSE* values determined by GA.

When different NLPCs are used as inputs, the result almost converges at the 40th generation in each case. Figure 9 shows the calculation process when three NLPCs are used as inputs.

3. FOA

FOA can find global optimization based on the food finding behavior of the fruit fly [31]. The fruit fly itself is superior to other species in sensing and perception.

An initial fruit fly swarm is generated randomly. Different directions and distances are set for searching for food via osphresis organs for this swarm. Since the food location cannot be known at first, the distance between the fruit fly and the origin of coordinates (D_i_) is first estimated, and the judgment value of the smell concentration (S_i_) is calculated. Here S_i_ is the combination value of the hyperparameters (C, ε, σ). S_i_ is substituted into the fitness function, to find the best smell concentration S_i_ of each fruit fly.

(21) $$Smell_{i}=f(S)=MSE_{CV}=\frac{\sum_{i=1}^{m}{\left(y_{i}-{\bar{y}}_{i}\right)}^{2}}{m}$$

where ${\bar{y}}_{i}$ is the training output and $y_{i}$ is the expected output.

**Figure 10:**
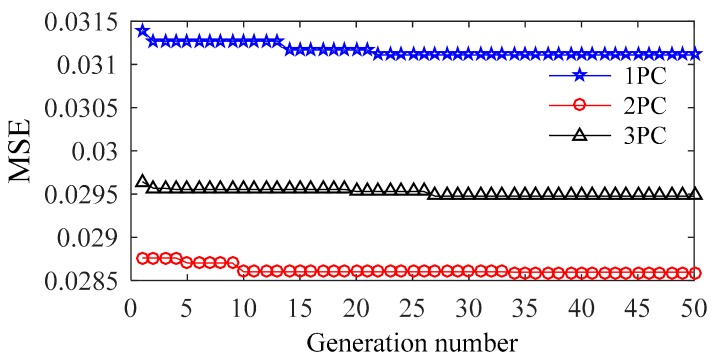
*MSE* values determined by FOA.

As there is no parameter to be set, FOA can easily be operated. The numbers of the generation and population are set at 50 and 20, respectively. Figure 10 shows the optimization process, different NLPCs are used as inputs. The results almost converged at 20th generation in each case.

### 4.3. Original Data-SVR Model

Using the original measured ambient data as the input, the settings for optimizing the hyperparameters using GSM, GA, and FOA are the same as in the previous section.

#### Optimization of hyperparameters

1. GSM

**Figure 11:**
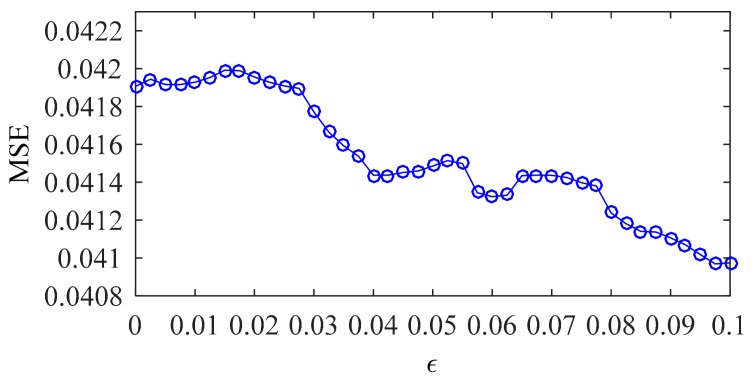
Iterative optimization process of GSM.

Figure 11 shows the optimization process for GSM. When ε equals 0.0975, the MSE reaches the minimum (0.040969) and the corresponding C and σ equal 0.08793 and 11.3137, respectively.

2. GA

**Figure 12:**
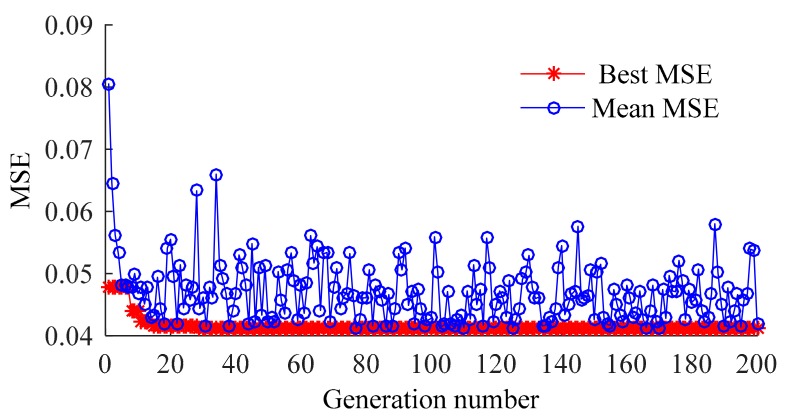
Iterative optimization process of GA.

Figure 12 shows the optimization process of GA. When C, ε, σ equal 59.5333, 0.092672, and 0.06361, respectively, the result converges at the 20th generation (MSE = 0.041225).

3. FOA

**Figure 13:**
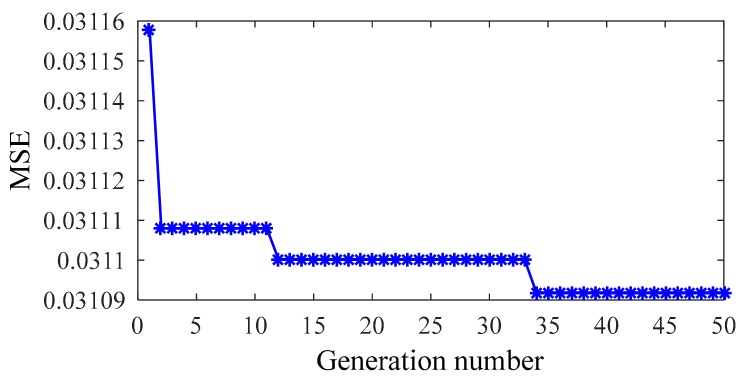
Iterative optimization process of FOA.

Figure 13 shows the optimization process of GA. When C, ε, σ equal 0.94248, 0.10587, and 0.1067, respectively, the result converges at the 20th generation (MSE = 0.031023).

### 4.4. Optimization Results

The optimization results of the two models are shown in Table 2 and Table 3. In comparing the optimal MSE values of the two models, the results of the NLPCA-SVR model are better than those of the OriginalData-SVR model. NLPCA-SVR is better to model the correlation between ambient factors and the modal frequency; when the first two NLPCs are used as inputs, the minimum value of MSE can be obtained and the FOA optimization result is the best of the three methods; in the 2PC-NLPCA-SVR model, the variation of the insensitive coefficient ε is small and the variations of the penalty parameter C and the kernel parameter σ are relatively large.

## 5. Estimation of the NLPCA-SVR Model

For SHM, generally the lower modal frequencies are applied. In this study, the first four modal frequencies are selected for data fitting (training sample) and predicting (testing sample). To provide more quantitative performance indicators, the performance of the NLPCA-SVR model is further tested. The generalization performance is evaluated by the hypothesis test and goodness-of-fit test.

### 5.1. Generalization Performance

The 2PC-NLPCA-SVR model is built from the first two NLPCs of ambient factors and the first 4 modal frequencies. Both the generalization performance rates for the training sample and testing sample are tested. A residual $e_{i}$ is defined to verify the generalization performance of the model. $e_{i}$ can be expressed as $e_{i}=y_{i}-{\bar{y}}_{i}$, where $y_{i}$ and ${\bar{y}}_{i}$ are the predicted value and the mean value of the modal frequency, respectively.

**Figure 14:**
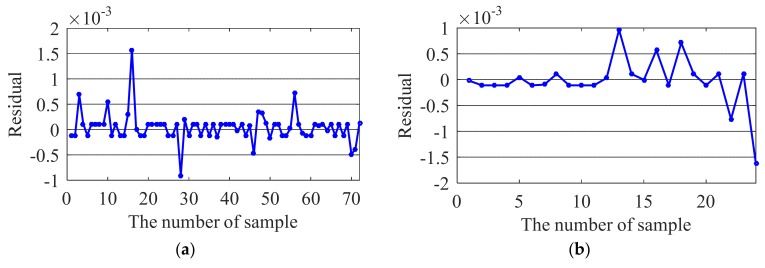
Residual of the first modal frequency: (**a**) Training sample; (**b**) Testing sample.

**Figure 15:**
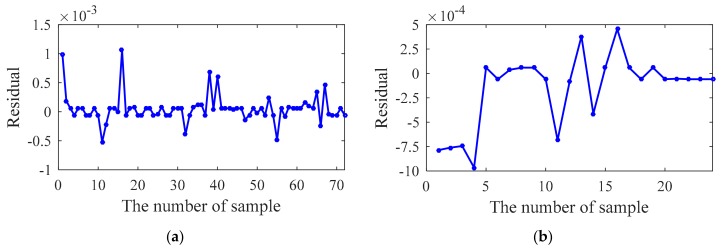
Residual of the second modal frequency: (**a**) Training sample; (**b**) Testing sample.

**Figure 16:**
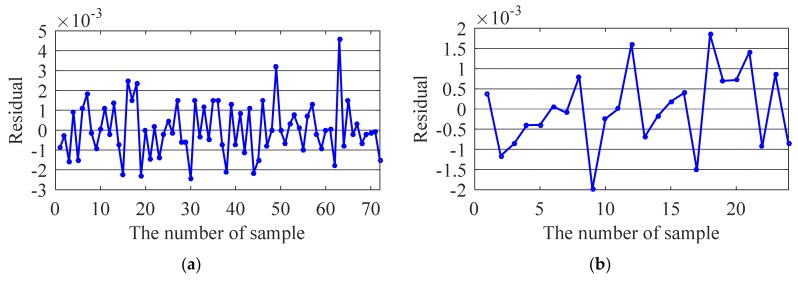
Residual of the third modal frequency: (**a**) Training sample; (**b**) Testing sample.

**Figure 17:**
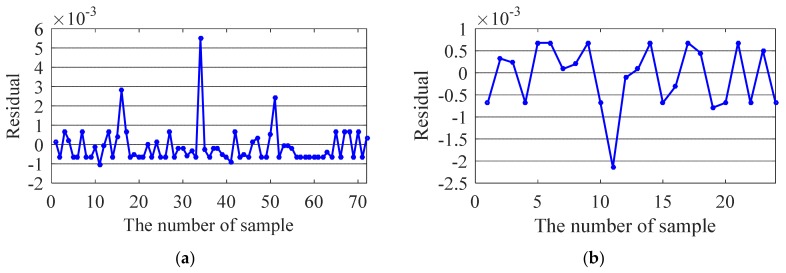
Residual of the fourth modal frequency: (**a**) Training sample; (**b**) Testing sample.

The test results are shown in Figure 14, Figure 15, Figure 16 and Figure 17. For the training samples, all the residuals are in the range [−2,2] × 10^−3^, [−2,2] × 10^−3^, [−5,5] × 10^−3^ and [−2,8] × 10^−3^; for the test sample, the residual ranges of the first three modal frequencies are within [−2,2] × 10^−3^. However, the prediction accuracy of the 4th frequency is slightly lower ([−6,2] × 10^−3^). In general, the correlation between the ambient factor and modal frequency can be closely reflected by the proposed method.

### 5.2. Hypothesis Test

To determine the generalization performances of the training and testing samples, a hypothesis test is performed on the 2PC-NLPCA-SVR model with the T test.

The T test is a testing method commonly applied for datasets with limited samples (less than 30). The mean *µ* and standard deviation *s* of the residuals *e_i_* in the regression model are defined as

(22) $$\mu =\frac{1}{m}\sum_{i=1}^{m}e_{i}$$

(23) $$s=\sqrt{\frac{1}{m}\sum_{i=1}^{m}e_{i}^{2}}$$

where m is the number of the sample.

The T-test steps for the residuals are as follows

(1) Hypothesis

(24) $$H_{0}:\mu =0,H_{1}:\mu \neq 0$$

Bilateral test and the level of the test (significance level α=0.05)

(2) Computing statistics and evaluating the value of t follows

(25) $$t=\frac{\mu -0}{s/\sqrt{m}}$$

(3) The value of T can be found according to the degree of freedom *df* = *m* − 1, and the calculated value t and the critical value T can be obtained.

In Table 4 and Table 5, for the significance level α=0.05, $\;\left|t\right|<T$, H_0_ is accepted, which means no significant statistics for the difference between the mean value of the samples and µ = 0.

### 5.3. Goodness-of-Fit Test

In statistics, an effective regression model can be divided into two basic components: the deterministic portion and the stochastic error. The former contains all the interpretable and predictable information in the regression model; the latter contains the random and unpredictable information. By building a regression model, the regular information is expressed in the form of functions and the irregular white noise (residual) is filtered out. When the residuals follow Gaussian distributions, the model extracts all available information.

**Figure 18:**
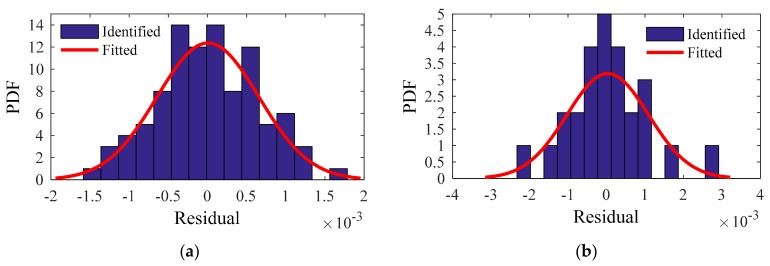
First modal frequency: identified PDF and fitted PDF of the residual: (**a**) Training sample; (**b**) Testing sample.

**Figure 19:**
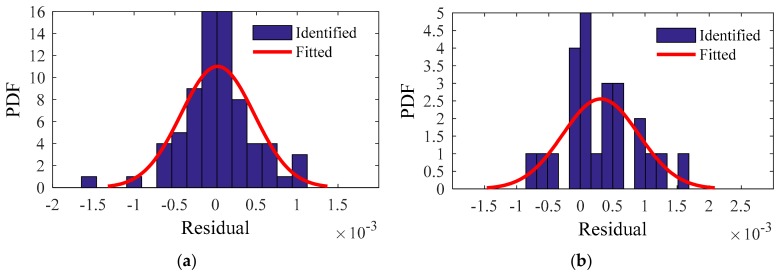
Second modal frequency: identified PDF and fitted PDF of the residual: (**a**) Training sample; (**b**) Testing sample.

**Figure 20:**
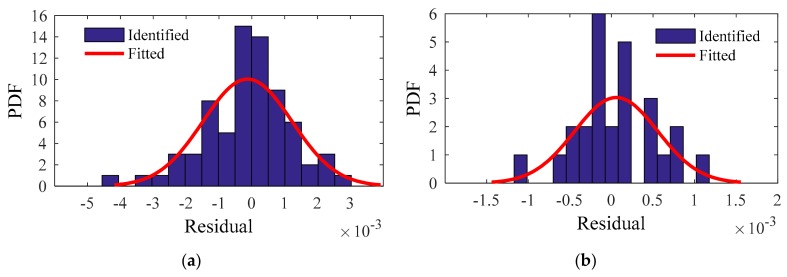
Third modal frequency: identified PDF and fitted PDF of the residual: (**a**) Training sample; (**b**) Testing sample.

**Figure 21:**
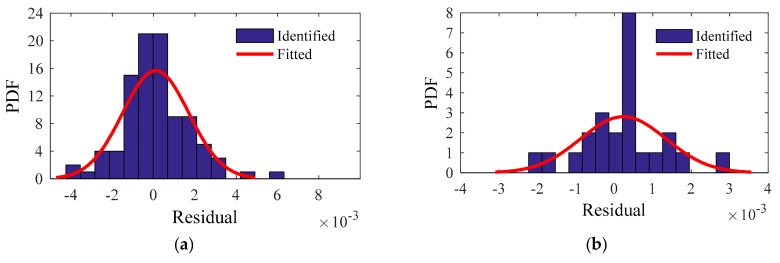
Fourth modal frequency: identified PDF and fitted PDF of the residual: (**a**) Training sample; (**b**) Testing sample.

For the residuals of the training and testing samples, the identified PDF and fitted PDF are shown in Figure 18, Figure 19, Figure 20 and Figure 21. The PDFs basically obey a normal distribution, which means no useful information in the residuals.

### 5.4. Updated Modal Frequency

The effects of ambient factors can be eliminated by Equation (26), and the updated modal frequency ${\hat{f}}_{i}^{t}$ can be expressed as

(26) $${\hat{f}}_{i}^{t}={\bar{f}}_{i}+f_{i}^{t}-f_{i}^{t}(S)$$

where ${\bar{f}}_{i}$ is the mean value of the ith modal frequency over a long period of time; $f_{i}^{t}$ is the identified value of the ith modal frequency at t moment; $f_{i}^{t}(S)$ is the predicted value of the ith modal frequency from NLPCA-SVR model.

**Figure 22:**
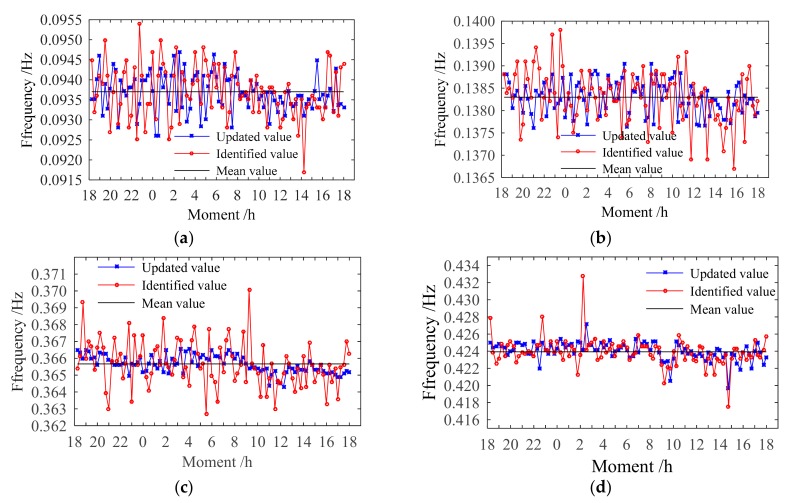
First 4 modal frequency changes after eliminating the influence of ambient factors: (**a**) First; (**b**) Second; (**c**) Third; (**d**) Fourth modal frequencies.

**Figure 23:**
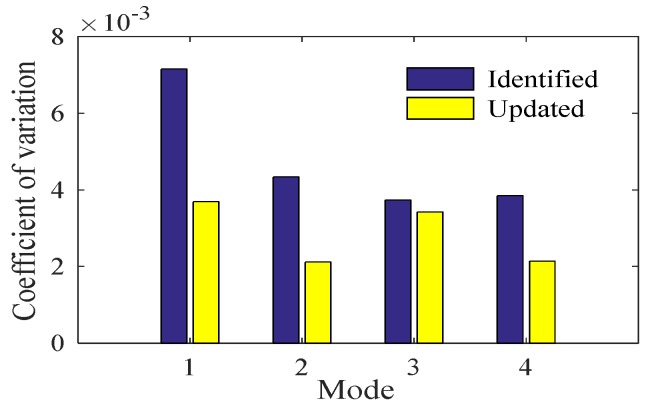
Variation coefficients of the first four modal frequencies.

Figure 22 shows the final results of the first four modal frequencies. The magnitude of the frequency change for each mode is obviously reduced, closer to the mean value of the identified modal frequencies. In Figure 23, the variances of the updated modal frequencies are lower than those of the identified modal frequencies. The maximum variance of the updated modal frequencies is 3.9 × 10^−3^, while the maximum variance of the identified modal frequencies is 7.8 × 10^−3^. The proposed method can thoroughly eliminate the effects of ambient factors.

## 6. Conclusions

This study proposed a NLPCA-SVR model to interpret the relation between ambient factors and modal frequencies. The following conclusions are summarized from the above study.

As the strong correlation among different ambient factors can be eliminated by NLPCA, the NLPCA-SVR model shows a superior generalization performance compared to the Original Data-SVR model. Though the monitoring period of the GNTVT benchmark only lasts for 24 h, the generalization performances of the NLPCA-SVR model is proved to be good while the amount of data is small. The minimum MSE can be obtained with the first two NLPCs, and the best combination of hyperparameters can be easily obtained using FOA. According to the equation proposed to update the modal frequency, the first four updated modal frequencies are closer to the mean value of the identified frequencies and the correlation variances are smaller. The maximum variance of the updated modal frequencies is 3.9 × 10^−3^, while the maximum variance of the identified modal frequencies is 7.8 × 10^−3^. The proposed method can thoroughly eliminate the effects of ambient factors. The NLPCA-SVR model is superior to other models in terms of its generalization capability and computational efficiency. While the data of ambient factors are big, the method of preprocessing the data with PCA and NLPCA cannot only ensure the fitting and generalization performance of the SVR model, but also reduce the computation and improve the calculation efficiency.

Meanwhile, the proposed method has been verified using the monitoring data only from 24 h monitoring of GNTVT under a specific variation of environmental temperatures. The variations in ambient factors were relatively small. To further validate the generalization capability of the NLPCA-SVR model, it is desirable to apply different bridges or buildings located in different regions with different weather conditions.

## Figures and Tables

**Table 1:** Correlation coefficients.

Data	Item	Correlation Coefficient		
Original ambient data	-	Temperature	Wind speed	Wind direction
	Temperature	1	0.2547	−0.3090
	Wind speed	0.2547	1	−0.2736
	Wind direction	−0.3090	−0.2736	1
NLPCs	-	NLPC1	NLPC2	NLPC3
	NLPC1	1	−2.46 × 10^−17^	5.66 × 10^−17^
	NLPC2	−2.46 × 10^−17^	1	9.73 × 10^−17^
	NLPC3	−5.66 × 10^−17^	9.73 × 10^−17^	1

**Table 2:** Optimal results of the NLPCA-SVR model.

Input	Method	Parameter			Optimal MSE
		C	σ	ε	
1NLPC	GSM	2.8284	8.8323	0.0975	0.040094
	GA	26.5678	4.7221	0.0973	0.040131
	FOA	1.1628	0.1197	0.1057	0.031218
2NLPC	GSM	0.0884	16.0739	0.1000	0.037817
	GA	0.3343	10.5849	0.1028	0.036574
	**FOA**	**0.8325**	**0.0938**	**0.0882**	**0.028612**
3NLPC	GSM	0.0625	11.3137	0.1000	0.038008
	GA	0.1855	7.6122	0.1157	0.036829
	FOA	0.9828	0.1168	0.1060	0.029503

**Table 3:** Optimal results of the OriginalData-SVR model.

Input	Method	Parameter			Optimal MSE
		C	σ	ε	
Original measured data	GSM	0.08793	11.3137	0.0975	0.040969
	GA	59.5333	0.06361	0.0927	0.041225
	FOA	0.94248	0.1067	0.10587	0.031023

**Table 4:** Hypothesis test results of the training sample.

Mode	µ	s	df	t	T
1	−4.7481 × 10^−5^	2.9386 × 10^−4^	71	−1.3710	1.993
2	−5.1555 × 10^−5^	2.4383 × 10^−4^	71	−1.7941	1.993
3	−3.0228× 10^−5^	1.3211 × 10^−3^	71	−0.1914	1.993
4	1.1689 × 10^−5^	9.6769 × 10^−4^	71	1.0249	1.993

**Table 5:** Hypothesis test results of the testing sample.

Mode	µ	s	df	t	T
1	2.6099 × 10^−5^	4.7299 × 10^−4^	23	0.2703	2.069
2	1.5349 × 10^−4^	3.7102 × 10^−4^	23	2.0266	2.069
3	1.5427 × 10^−5^	9.6760 × 10^−4^	23	0.0781	2.069
4	8.9497 × 10^−5^	7.1578 × 10^−4^	23	0.6125	2.069
